# Serum Vitamin D Level among Multiple Sclerosis Patients in the Tropics: Experience from a Private Clinic in Addis Ababa, Ethiopia

**DOI:** 10.4314/ejhs.v31i3.18

**Published:** 2021-05

**Authors:** Biniyam A Ayele, Mehila Z Wuhib, Betesaida G Zenebe, Guta Z Metaferia

**Affiliations:** 1 Department of Neurology, School of Medicine, College of Health Sciences, Addis Ababa University; 2 Yehuleshet Specialty Clinic, Addis Ababa, Ethiopia; 3 Department of Neurology, School of Medicine College of Health Sciences, Addis Ababa University, Addis Ababa, Ethiopia

**Keywords:** Multiple sclerosis, hypovitaminosis D, latitude, vitamin D, Ethiopia

## Abstract

**Background:**

Multiple sclerosis (MS) is an immune mediated disabling neurological disorder. Very little is known about MS in Ethiopia. The objective of this study was to determine the prevalence of hypovitaminosis D and associated factors in cohort of MS patients in Ethiopia.

**Method:**

A cross-sectional observational study was conducted among 25 multiple sclerosis patients at Yehuleshet Specialty Clinic, Addis Ababa, Ethiopia.

**Results:**

The prevalence of vitamin D deficiency was 96% (n=24). The average serum vitamin D was 14.8 (±10.4) ng/mL. The mean age was 35.8 (±10) years. Females accounted for 80% (n=20). Relapsing and remitting MS was the commonest variant. Motor, sensory, and mixed symptoms accounted for 40% (n=10), 20% (n=5), and 24% (n=6), respectively. Cold or hot weather and stress were reported as worsening factors in 24% (n=6). Relapse rate was 44% (n=11). Fatigue and seizure disorder were reported by 80% (n=20) and 16% (n=4) respectively. Steroid is the commonest prescribed medication for the patients. A negative correlation was found between serum vitamin D and age (r = -0.062, p = 0.7). Similarly, a negative association was observed between vitamin D and duration of illness (r = -0.311, p = 0.1). Fatigue was reported by those with moderate hypovitaminosis compared to those having severe hypovitaminosis (p=0.002). Seizure was reported more by those with vitamin D below 10ng/mL compared to those having above 10 ng/mL (p=0.004).

**Conclusion:**

Our study demonstrates a high prevalence of hypovitaminosis D in Ethiopian MS patients. Hypovitaminosis D was associated with increment in age and duration of illness.

## Introduction

Multiple sclerosis (MS) is an immune-mediated central nervous system (CNS) disorder, characterized by demyelination, chronic inflammation, oligodendrocyte death, and axonal loss. It affects young females disproportionally compared to males (1). The etiology of MS may be multifactorial, possibly involving genetics, environmental factors, infections, latitude gradient, and vitamin D deficiency (2). In the past two decades, a plethora of scientific evidences based on epidemiological and genetic studies reported an association between low serum vitamin D concentration and multiple sclerosis. According to a recently published review article, the highest prevalence was observed in Australianborn Australians and the lowest prevalence was seen in Black African (3).

The geographic latitude of Ethiopia at slightly more than 9 degrees (ranging from just over 3 degrees at Moyale to almost 14 degrees at Mekele) makes it a low prevalence region for MS with an estimated prevalence of 1–5/100,000 people (3). The first case of multiple sclerosis was reported from Ethiopia 35 years ago (4). Subsequent improvement in diagnostic capabilities including high resolution MRI coupled with an increasing number of welltrained neurologists have unquestionably contributed to the diagnosis and management of MS in Ethiopia. Recently published studies demonstrated a high prevalence of vitamin D deficiency among the Ethiopian population despite the tropical location with abundant sunlight (5,6). Furthermore, the vast genetic diversity observed in Ethiopian population and high prevalence of hypovitaminosis D may be contributing to the current epidemiological shift observed in increasing number of multiple sclerosis cases. Contrary to the general belief among most scientists that multiple sclerosis is rare among black Africans and population living in the tropics, we believe this study will shade some light on this disabling immune-mediated neurological disorder.

The objective of this study was to assess the prevalence of vitamin D deficiency and associated factors among multiple sclerosis patients in Ethiopia. To the best of our knowledge, this is the first study to report on multiple sclerosis and vitamin D status in Ethiopia.

## Materials and Methods

**Study objective and study setting**: The aim of our study was to assess serum vitamin D level and associated factors in patients with multiple sclerosis. The study was conducted at the Yehuleshet Specialty Clinic (YSC) Outpatient Neurology Clinic located in the heart of Addis Ababa, Ethiopia. YSC provides comprehensive care for a large number of neurology patients and is fully equipped with MRI, EEG, NCV/EMG, and evoked potential testing as well as routine non-neurological services such as hematologic and other laboratory studies, x-rays, ECG, and ultrasound. Ethiopia lies between the Equator and Tropic of Cancer, between the 3° N and 15° N Latitude and 33° E and 48° E Longitude. The capital, Addis Ababa, is located at the foot of Entoto Mountain, 3000m above sea level (7).

**Study period and design**: The study was a cross sectional observational survey conducted at YSC between November 2019 and April 2020. All MS patients aged 18 years and above were included in the study. The diagnosis of MS was made by certified neurologists (BAA, MZW, and GZM) based on the 2017 Revised McDonald Criteria (8), verbally consented to participate, and serum vitamin D levels were included in the study. The following reference standards were used during analysis for serum Vitamin D level: Severe deficiency (< 12ng/ml), moderate deficiency (12–20 ng/ml), mild deficiency (20–30 ng/ml), and normal (> 30 ng/ml) (9).

**Data collection tool and procedure**: A structured questionnaire was used in assessing the socio-demographic, serum vitamin D, clinical characteristics, and type of MS, treatment, and comorbid disorders. All 25 MS patients included in the study were evaluated, and each patient was examined by one of three board-certified neurologists. Patients' medical recorders were reviewed for additional clinical data. All patients were diagnosed with MS and followed at YSC from November 2019 through the present.

**Data analysis**: Completed questionnaires were cleaned and entered into Statistical Package for Social Sciences (SPSS) Version 25 for analysis. We used descriptive statistics with frequency and proportion for categorical data and mean, range, and standard deviation (SD) for continuous variables. Associations between selected variables were tested using Student's t-test and Fisher's exact or Chi-square tests. Variables with p values <0.05 were considered statistically significant.

## Results

**Sociodemographic characteristics and duration of illness**: The average age of study participants was 35.8 (±10) years (Range: 19–60 years). Half of the patients were aged between 30 and 40 years. Females accounted for 80% (n=20). The average duration of illness was 3.4 (±3.2) years (Range: 1 – 11 years). The majority (n=9, 36%) of them were government employees, while 8% were students. Seven (28%) of the study participants changed their jobs after being diagnosed with MS. Twenty-one (84%) of the patients attended grade 12 and above (Table 1).

**Table 1:** Sociodemographic characteristics and duration of illness of study participants

Characteristics		Number (%)
Age category	≤ 30 years	7 (28%)
	31 – 40 years	13 (52%)
	> 40 years	5 (20%)
Gender	Male	5 (20%)
	Female	20 (80%)
Duration of illness	≤ 5 years	18 (72%)
	>5 years	7 (28%)
Change of job due to illness	Yes	7 (28%)
	No	18 (72%)
Educational level	Grade 7 – 12	4 (16%)
	Grade 12 and above	21 (84%)
Occupation	Private business	7 (28%)
	Government employee	9 (36%)
	Daily laborer	1 (4%)
	Housewife	5 (20%)
	Students	2 (8%)
	Non-governmental organization	1 (4%)

**Vitamin D level, classification and clinical characteristics of study participants**: Twenty-four (96%) of the study participants had vitamin D deficiency (< 30ng/mL). The mean serum vitamin D was 14.8 (10.4) ng/mL (range: 4 – 53.1 ng/mL). Nearly half (48%) had serum vitamin D < 10ng/mL. All patients with hypovitaminosis D treated with standard regimen of vitamin D supplementation, advised on importance of sunshine exposure, and nutritional advice. On subsequent follow up, serum vitamin D was determined for ten (40%) of them. The average serum vitamin D was 47.5 (±27.7) ng/mL. All the ten patients reported mild to moderate improvement in their initial presenting symptoms. Among a total of 25 MS patients, 56% had relapsing and remitting MS (RRMS), 16% had primary progressive MS (PPMS), and 20% had clinically isolate syndrome. The commonest presenting symptoms are motor (40%), followed by mixed clinical features (sensory + motor + visual) and sensory features (20%).

Nearly half (44%) of our study participants reported one or more relapse after their initial diagnosis. Meanwhile, 80% of the patients reported fatigue, and 16% had seizure (Table 2). Among additional symptoms, ten (40%) had gait abnormality, 12% had bowel/bladder dysfunction, and 12% reported pain. Six (24%) reported cold or hot weather and stress worsened their symptoms. Funduscopic examination showed optic neuritis in 16%, optic atrophy in 8%, and normal in 76%.

**Table 2:** Serum vitamin D, classification and clinical characteristics of study participants

Characteristics		Number (%)
Vitamin D level	≤ 10 ng/ml	12 (48%)
	11 – 20 ng/ml	8 (32%)
	>20 ng/ml	5 (20%)
Classification of MS	Relapsing and remitting MS	14 (56%)
	Primary progressive MS	4 (16%)
	Secondary progressive MS	2 (8%)
	Clinical isolate syndrome	5 (20)
Presenting symptoms	Sensory features	5 (20%)
	Motor features	10 (40%)
	Visual complaints	4 (16%)
	Mixed features	6 (24%)
Relapse	Yes	11 (44%)
	No	14 (56%)
Fatigue	Yes	20 (80%)
	No	5 (20%)
Seizure disorder	Yes	4 (16%)
	No	21 (84%)

¶MS: Multiple sclerosis

**Figure 1:**
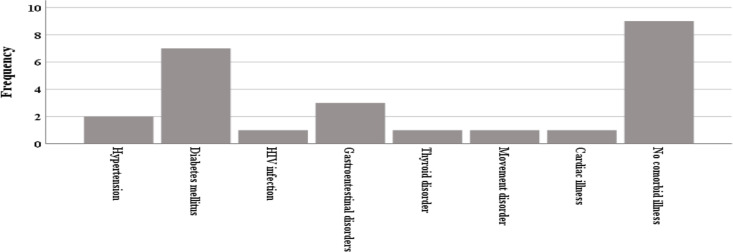
Distribution of comorbid illnesses of the study participants

**Treatment, comorbidities, and associated factors among study participants**: Three of our patients received disease modifying drugs for MS treatment. Two received Rituximab and one received Azathioprine. Seventeen (68%) patients were treated with steroid at first attack or relapse. Among reported comorbid illnesses, diabetes mellitus (DM) accounted for 28%, HIV infection accounted for 4% and 36% reported no comorbid illness (Figure 1). Four (16%) reported history of alcohol ingestion, 20% reported having previous history of head injury, and one patient was tested positive for syphilis. None of our patients reported family history of MS and cigarette smoking. Trigeminal neuralgia was diagnosed in 8% of our study participants.

**Figure 2:**
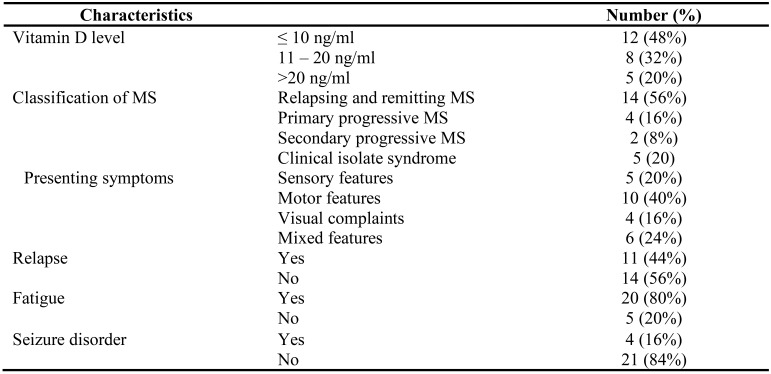
Scatter plots showing negative association between serum vitamin D and age.

**Association between serum vitamin D, age, and duration of illness**: Pearson's correlation was done to determine possible association between serum vitamin D and age and duration of illness. The present study showed negative correlation between serum vitamin D level and age (r = -0.062, p = 0.7, R^2^= 0.004) (Figure 2).

**Figure 3:**
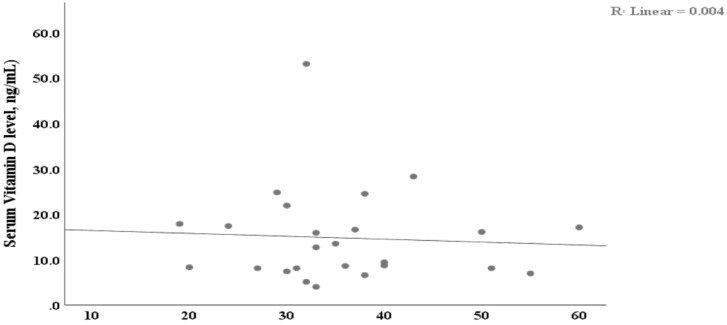
Scatter plots showing negative association between serum vitamin D and duration of illness.

Similarly, negative association was observed between duration of illness and serum vitamin D (r = -0.311, p = 0.1, R^2^= 0.097) (Figure 3). Furthermore, age and duration of illness were responsible for 0.4% and 9.7% of the variation (R^2^) observed in serum vitamin D level.

**Association between hypovitaminosis D, fatigue, and seizure disorders**: Fisher's Exact Test was done between serum vitamin D and fatigue and between vitamin D and presences of seizure disorder. Statistically significant correlation was observed between fatigue and serum vitamin D level > 10ng/mL (p = 0.02). Those MS patients having moderate vitamin D deficiency (> 10ng/mL) reported fatigue more compared to those having level < 10ng/mL because, less proportion of patients had level < 10ng/mL (48%) compared to level > 10ng/mL (52%) (Table 1). Seizure disorder was associated with hypovitaminosis D (p = 0.04) (Table 3).

**Table 3:** Association between hypovitaminosis D level and different variables

		Serum Vitamin D level		Fisher's Exact Test CI 95%
		
		≤ 10 ng/mL N (%)	> 10 ng/mL N (%)	
Fatigue	Yes	7 (28%)	13 (52%)	**P = 0.02**
	No	5 (20%)	0 (0.0%)	
Seizure	Yes	4 (16%)	0 (0.0%)	**P = 0.04**
	No	8 (32%)	13 (52%)	
Gender	Male	1 (4%)	4 (16%)	P = 0.3
	Female	11 (44%)	9 (36%)	
Relapse	Yes	7 (28%)	4 (16%)	P = 0.2
	No	5 (20%)	9 (36%)	
Type of MS				
	Clinically isolate syndrome	2 (8%)	3 (12%)	P = 0.3
	Relapsing and remitting MS	9 (36%)	5 (20%)	
	Secondary progressive MS	0	2 (8%)	
	Primary progressive MS	1 (4%)	3(12%)	

¶MS: Multiple sclerosis

## Discussion

High prevalence of vitamin D deficiency (96%) was found among Ethiopian MS patients with mean (±SD) of level 14.8 ± 10.4ng/mL. These findings were in agreement with previous reports of low vitamin D among Ethiopians, despite abundant sun light (5,6). Females accounted for majority of MS and hypovitaminosis D (1,10–12). Steroid is the most commonly prescribed medication, while a minority of the patients received disease modifying therapy. Furthermore, inverse correlation was found between serum vitamin D level and age, as well as between vitamin D level and duration of illness A significant proportion of our patients reported fatigue (13). Fatigue and seizure were in significant statistical agreement with low serum vitamin D level.

In our study mean age was 35.8 years, which is in line with similar reports (14–16). Half of our patients had severe vitamin D deficiency. These findings are consistent with previously reported studies (17–19). Relapsing and remitting MS is the predominant type of MS observed among our patients. Majority of the patients presented with symptoms related to disturbance of motor, sensory, and visual system. These findings were consistent with studies from Egypt and Europe (20–24). The observed low proportion of secondary progressive MS type in this study could be due to relatively short disease duration observed in our study. Although, multiple sclerosis is predominantly a demyelinating CNS disorder, cortical features such as seizure disorders are not uncommon. In our study four patients had seizures, which was associated with low serum vitamin D level (P = 0.04). Several studies reported prevalence of seizure disorder and epilepsy to range from 0.5% – 3% among MS patients (25,26).

The management of MS has changed significantly in recent decades. Currently patients with established MS are treated with Disease-modifying drugs (DMD) (20,23,25). In our study, only three MS patients received DMDs (Rituximab and Azathioprine) due to the unavailability of those medications in Ethiopia. The most common risk factors identified in our study were vitamin D deficiency and female gender.

A small number of our patients had a history of either alcohol ingestion or head injury, but none of the patients used tobacco. There was no relevant family history for any of the patients (22,27–31). The prevalence of comorbid, diabetes mellitus, hypertension, and HIV infection was 28%, 8%, and 4% respectively. These findings were supported by similar studies (32,33). These observations may be attributable to the ever increasing degree of immobility and fatigue observed among MS patients, prolonged steroid and DMD usage (68% in our study), and increasing life expectance predisposing MS patients to comorbid illnesses.

There was an inverse correlation between serum vitamin D levels and both the age distribution and duration of illness (Figures 2 and 3). These study findings indicates as the age and duration of illness increase serum vitamin D level will decrease, which could be attributable to disease related disability and aging hindering adequate sun exposure. These findings are supported by study by Fahmi et al from Egypt (10). Furthermore, 0.4% and 9.7% of variations observed in serum vitamin D level was due to variation related to age and duration of illness, respectively (R^2^= 0.004 & R^2^= 0.097 respectively). Hypovitaminosis D is believed to be associated with several other autoimmune disorders. Similarly, several in vitro and in vivo studies have shown that vitamin D has antiinflammatory effects by suppressing the innate as well as the adaptive immune system (16,18,34–36).

Thus, future researches should be done in order to identify other potentially modifiable factors responsible for the rest of 90% variation not attributable to age and duration of illness. Fatigue is reported to occur in 70%–90% of MS patients and is considered to be one of the most disabling symptoms at all stage of MS (34). Furthermore, hypovitaminosis D is commonly associated with fatigue (6). Our study found statistically significant association between suboptimal level of vitamin D and fatigue, which is consistent with similar studies (6,13). Limitations of our study includes: small sample size, lack of control group, lack of valid tool to assess fatigue, and failure to screen our patients for other potential causes of hypovitaminosis D. Furthermore, we have not assessed the Body Mass Index (BMI) of our study participants, even though obesity has a strong association with vitamin D deficiency.

In conclusion, our study demonstrates a high prevalence of hypovitaminosis D in Ethiopian multiple sclerosis patients. Hypovitaminosis D was associated with increment in age and duration of illness. Future controlled study is recommended to consolidate our findings. Thus, routine serum vitamin D levels determination maybe beneficial as hypovitaminosis D is one of potentially reversible metabolic disorder associated with multiple sclerosis. Furthermore, we recommend investigating the role of comorbid diseases such as DM in pathogenesis and progression of MS.

The study received ethical approval from City Government of Addis Ababa Health Bureau Ethical Clearance Committee (Protocol number: A/A/HB/437/227). All subjects provided written consent before conducting the interview.
